# A community-based intervention approach to control disease outbreaks and climate-related deaths in communally raised goat kids in the Eastern Cape Province, South Africa

**DOI:** 10.1007/s11250-022-03143-5

**Published:** 2022-03-22

**Authors:** Mhlangabezi Slayi, Leocadia Zhou, Thobela Louis Tyasi, Ishmael Jaja

**Affiliations:** 1 Risk and Vulnerability Science Centre, University of Fort Hare, Alice, South Africa; 2 Department of Agricultural Economics and Animal Production, University of Limpopo, Polokwane, South Africa; 3 Department of Livestock and Pasture Science, University of Fort Hare, Alice, South Africa

**Keywords:** Animal health, Community engagement, Diseases, Extension services, Intervention model, Resource-constrained farmers, South Africa

## Abstract

A community-based intervention project was conducted, from April 2017 to March 2019, on 512 kids born from flocks of 30 purposively selected households located in ten villages within Alice district of the Eastern Cape Province, South Africa. The study aimed to examine the effectiveness of combined efforts from the research team and farmers to control disease outbreaks and climate change-related deaths. A systematic dosing and fortnight dipping schedule was part of the intervention efforts made by the research team as well as supplying feed to pregnant and lactating does. Proper housing shelters were constructed and practicing hygiene measures were implemented. Veterinary assistance and medication were availed whenever there was a sick kid. Diseases and climate-related deaths were diagnosed based on clinical signs, laboratory results and relevant necropsy records. The current intervention program resulted to a decline in kid mortality rate (56.17% to 22.38%). Consistent access to veterinary services reduced the prevalence of Infectious diseases in year-2 (6.38%) as opposed to year-1 (14.89%). Climatic factors (11.92 vs 2.89%) became less prevalent due to better housing infrastructure while parasitic-related health problems showed a similar trend (9.79% vs 1.81%) after implementing a systematic dosing plan and fortnight dipping schedule. Death due to mechanical (7.66% vs 3.97%), reproductive (5.53% vs 3.25%) and nutritional (6.38% vs 2.53%)-related health problems also showed a slight decline. Even though the mortality rate was still above 20%, the documented improvement in kids’ survival rate implies that the approach was a moderate success. An in-depth analysis with regard to affordability and effectiveness should be conducted to ensure consistent support.

## Introduction

Goats are the most popular livestock species kept by farmers in South Africa (Dube et al., 2016). A high proportion of them in the country is found in rural areas of the Eastern Cape Province (Idamokoro & Masika, 2017). More than three-quarters of the goat population in the province is reared under traditional farming systems (Slayi et al., 2014). Goat rearing in rural communities is mostly practiced by resource-constrained farmers who barely afford conventional drugs and solely rely on traditional medicine to cure various ailments and on natural resources as a source of feed and access to drinking water (Khandoker et al., 2018). Communal farmers prefer to rear goats for their low cost of production, prolificacy, and excellent adaptive capacity to warm environment, dynamic feeding behaviour and fast reproduction cycle (Sebei et al., 2004). Goat rearing plays a prominent role in the livelihoods of many small and marginal farmers, including landless labourers (Faruque et al., 2017). Socially, goats serve as a source of protein in human diets during festival gatherings and provide good and stable source of income, especially for the underprivileged people in rural areas (Abiola & Onwuka, 2021; Wachida et al., 2018). The degree to which goats survive to marketable age is one of the prominent indicators for efficient goat production (Mhlanga et al., 2018). Evidence show that goats serve as saving and living banks for the financially disadvantaged rural people since they can easily be converted into cash when a need arise by virtue of their ready market demand (Moni & Samad, 2019). In addition, goat rearing has been modelled by various government and non-governmental research institutions all over the world to mitigate rural poverty (Yasmin et al., 2020).

Annually, a high number of goats is sold during the winter and summer months as well as during Easter holidays of the year, where most of the traditional ceremonies and rituals are likely to be performed (Idamokoro & Masika, 2017). Developing countries like South Africa have substantial potential for animal production (Donkin & Boyazoglu, 2004), and several reports have shown a growing demand for domestic goat products (Dube et al., 2016). Increased consumer demand could be attributed to popularised health benefits of goat products, as recommended by many researchers and health awareness professionals (Diriba & Kebede, 2020). Among those health benefits is that health conscious consumer groups accept meat and dairy products derived from goat milk due to its low contents of allergens known to be responsible for several health risks in humans (Oyeyemi & Akusu, 2021). Despite this growing consumer demand and acceptance of goat products, there seem to be endless production hurdles impeding the marketing and availability of goat products, thus threatening the sustainability of the sector in South Africa(Slayi et al., 2014). Due to its overreliance on natural resources, goat production in resource-poor countries is showing increased vulnerability to climate change-related stressors such as extreme weather conditions, forage instability and frequent disease outbreaks (Snyman, 2010). Consequently, increased kid mortality rates have become a norm in communally raised goat flocks (Donkin & Boyazoglu, 2004). Anecdotal reports and recent literature claim that the reason to high mortality rate in young stock is often unknown and sometimes worsened by the frequently occurring climatic extremes and negligence in disease control measures in resource-poor countries throughout the world (Alemnew et al., 2020; Ebozoje & Ngere, 2021).

Mortality rates of kids from birth to weaning is one of the prime effects on the viability of goat production efficiency in communal areas (Perumal et al., 2019; Yasmin et al., 2020), with kid mortality rates as high as 46.51%, having been reported in various farming communities across South Africa (Sebei et al., 2004; Slayi et al., 2014). The untimely exits restrict the number of animals available for future replacement and impact the number of animals reaching marketable age (Chauhan et al., 2019). Increased kid mortality rate is sometimes attributed to genetic and environmental factors (Alemnew et al., 2020; Perumal et al., 2019). Insufficient milk production from the does has been cited as a major constraint directly linked to reduced total productivity. Among the factors contributing to postnatal kid mortality are birth type (single, twins or triplets), season, age, sex, birth weight and difficult or prolonged birth weight (Robertson et al., 2020). In a study conducted by Slayi et al. (2014), it was noted that farmers are quite aware of some of the leading causes of kid mortalities in communal goat flocks. However, insufficient attempts to validate allegations made by farmers continue to hamper the suboptimal performance and the ability to develop and apply management interventions to control disease prevalence and climate change-related deaths in rural communities. Furthermore, inadequate support from the government and private research institutes with respect to initiation and facilitation of control programmes or funding of research on diseases and climatic-related deaths affecting adult goats and their kids still poses another challenge to communal goat farming. Community-based intervention initiatives may be seen as potential solutions to improve the survival of kids, which later influences the viability of the goat enterprise through improved production efficiency and good financial returns. The current study was therefore conducted to determine the effectiveness of a community-based intervention program aimed to control the disease outbreaks and climate-related deaths impacting goat kids in selected villages within the Eastern Cape Province, South Africa.

## Materials and methods

### Description of experimental site

**Fig. 1:**
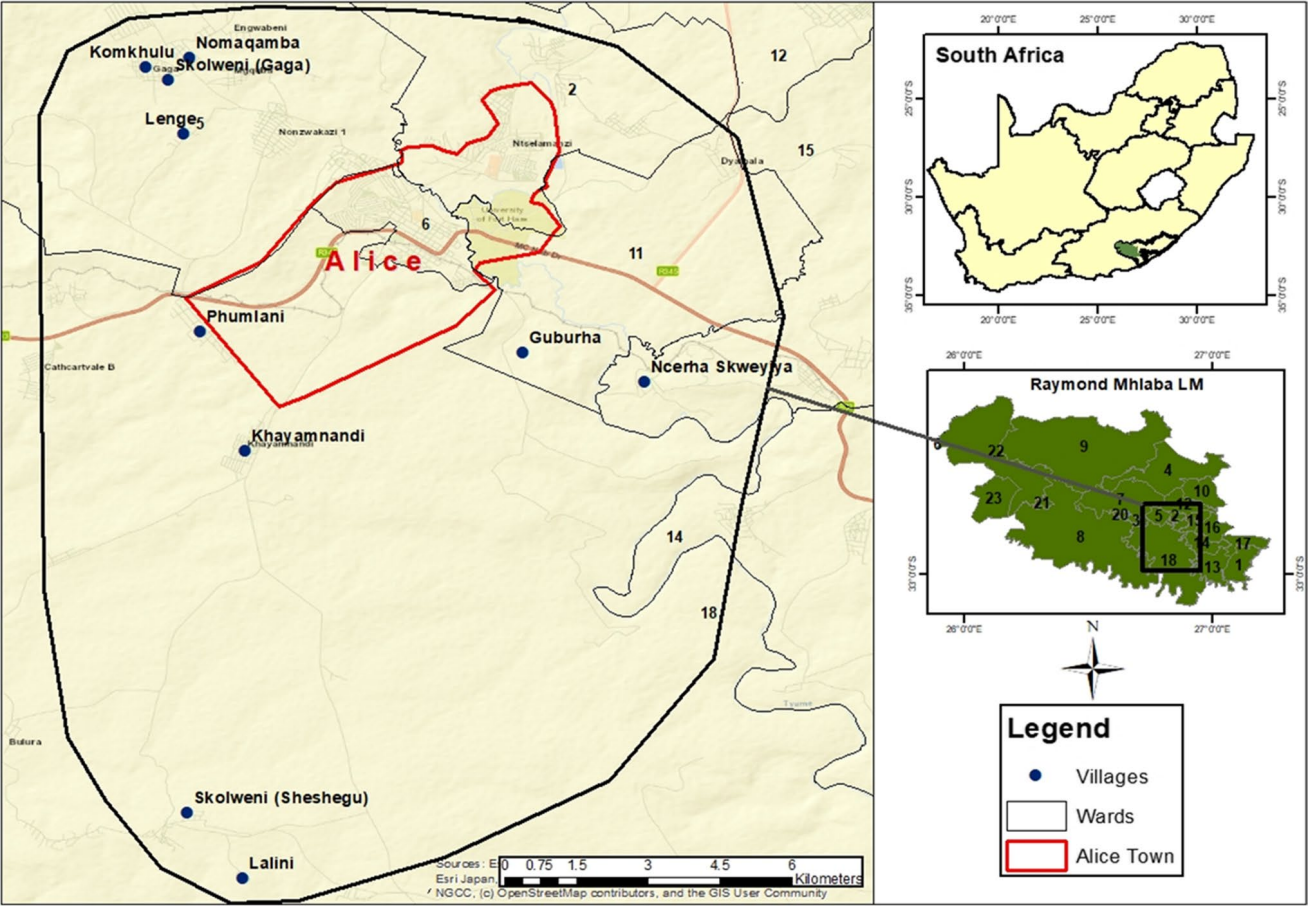
Geographic location of ten villages used study sites within Alice district under Raymond Mhlaba Local Municipality. Source: Adapted from Google earth

A community-based intervention trial to control diseases and climate-related deaths amongst village-owned goat kids was conducted in ten villages located within the Alice district in the Eastern Cape Province of South Africa from April 2017 to March 2019. The Eastern Cape Province is made up of thirty seven districts. The Alice district falls under Raymond Mhlaba Local Municipality in the Eastern Cape Province. The municipality covers 3725 km^2^, has an average of 43 people/km^2^ or 0.43 people/ha., and is made up of 21 wards and 194 villages. Alice district is about 21.97 km^2^ wide with an altitude of 522 m asl and is located 32^o^47′S26^o^50′E. The site is situated 80 km inland from the Eastern Cape coast line. Livestock production is the lifeblood of this resource-constrained local population keeping indigenous goat breeds as the most preferred livestock species in the area. The region is sparsely populated and fragile to climate variability experiencing extremes of drought and floods. Animals in the study area rely on natural pastures as a source of feed. The climate of the study area is characterized by a slightly hot summer, high humidity all the year-round and inconsistent rainfall (annual average rainfall of 473.2 mm), which is received during November to April. Averagely, the site recorded a daily maximum temperature of 25.8 °C, and a minimum temperature of 11.2 °C. Humidity is high throughout the year, the average being 72.1%. The study area is located in a hot, humid zone having four distinct seasons, viz., post-rainy (March to May), cold-dry (June to August), hot-dry (September to November) and hot-wet (December to February). The area lies in a lowland characterised by steep, isolated mountains, and the veld type is predominant of Bhisho Thornveld. Several trees characterise the vegetation in the region, including shrubs, and grass species with *Acacia karroo*, *Themeda triandra*, *Panicum maximum*, *Digitaria eriantha*, *Eragrostis* spp., *Cynodon dactylon* and *Pennisetum clandestinum* being the dominant plant species. Soils are extremely heterogeneous but are predominantly sedimentary (sand and mudstones) with some variation when intrusions of igneous rock (doleritic dykes and sheets) result in red soils occurring in some areas. Figure 1 shows the location of the villages used as sites during the field trial period.

### Ethical consideration and process of mobilizing farmers

Before entering the communities for actual data collection, a prior informed consent was obtained from the Research Ethics Committee of the University of Fort Hare (MAP021SSLA01). The quality of data to be collected in a participatory manner in traditional communities largely depends on the genuine cooperation of the community. This genuine cooperation stipulates clarification and sensitization of the community on the objectives, intentions and use of possible outcomes of the study. Accordingly, a series of community meetings were organized at each village before the commencement of actual field work. The headmen in each village were contacted and briefed on the rationale for conducting the meetings. This was done to explain in detail the procedure of the project, including its objectives and outcomes. The meetings were then called by and conducted in the presence of the headmen.

### Sampling of village participants and management of experimental animals

There are thirty seven district municipalities in the Eastern Cape Province of South Africa. From the existing district municipalities in the province, Alice district was purposively chosen because of its rich history of being known as a livestock-based community with a high goat population in the province. Villages that participated in the study were selected using a 30% random sampling criterion. Ten villages from the total number of 30 villages were chosen to participate in the study. The selected villages had the advantage of being close to the research team, allowing frequent visits during data collection period. Villages of interest were Skolweni (Sheshegu), Lalini (Sheshegu), Phumlani, Guburha, Khayamnandi, Lenge, Skolweni (Gaga), Komkhulu, Nomaqamba and Ncerha-Skhweyiya. Three flock owners were selected to participate in the study using a snow-ball sampling technique from each of the selected villages. In this way, thirty farmers were selected from 10 villages which constituted the sample. The method was chosen because it only selected farmers who rear goats as adjuncts to their main occupation and have a history of being known to experience high kid mortality rates in their flocks. This was the basis of the study. A total of number of 512 goat kids born within the selected flocks were subjects during the 2-year trial period. Newly born kids were weighed a few minutes after birth and housed separately in animal shelters for 3 months before joining the flock. The ages of the kids considered were from birth to 12 months; that is, animals closely monitored from birth until they died or were 1 year old or the study ended, whichever came first. In all, kids older than 1 year were no longer followed or censored at the end the study. All the kids born during the research period were ear-tagged to ensure easy identification during field visits and data gathering during the trial. During the day, flocks from all the participating villages browse (from 7 to 4 p.m.) on communal grazing areas dominated by open grassland. There was virtually no habit of supplementation of any group of goats, including nursing and pregnant does. Goats access drinking water from nearby streams and dams. The goats reproduce year-round without a controlled breeding program. During the first year of the study, flock owners were responsible for daily kid care and administering treatment of illnesses independently without any experimental treatments or interventions applied by the research team. In the second year of the trial, the flock health management was maintained through regular follow-up by the research team and treatment of clinical cases. These include regular deworming program based on coprological examination for internal parasite infestation as well as regular spray dipping for external parasites and vaccination for major bacterial and viral small ruminant diseases such as pneumonia, coccidiosis and other infectious diseases prevalent in the area was done.

### Data collection and research team participation

Diseases and climate-related deaths were documented over a period of 2 years (April 2017 to March 2019). Diagnosis of diseases and climate-related deaths was based on previous history recorded from farmers, clinical signs and laboratory findings. Data gathering was made possible through close cooperation between the farmers and the research team. To ensure the consistent commitment of farmers to the project and achieve the study objectives, regular flock visits were done throughout the follow-up period, and prior arrangements were made with the farmers. Data collected during the first year of the trial was used as baseline information to measure and understand the severity of the study problem. For effective measurements and accurate results, farmers were allowed to continue managing their animals without any interference from the research team regarding veterinary assistance, feed supply and extension services. While in the second year of the trial, intervention efforts were implemented by the research team. These include a dosing program and a dipping program for each farmer participating in the project. Housing shelters for kids were also constructed and farmers were instructed to ensure that the manure is always collected every month throughout the study period. Feed was supplied to each farmer and given to the pregnant and lactating does. Veterinary assistance and medication was availed to each farmer whenever there was a sick kid. Prior to collecting data, farmers voluntarily committed to informing the research team whenever there is a new born kid in the flock. This included calling the research team whenever there was a sick or dead kid. Upon arrival, the team recorded the date of birth, and birth weights were recorded. Whenever there was a sick or dead kid in their flock, the farmers contacted the research team. The cause of illness or death was determined from the clinical history, clinical observations, laboratory findings and relevant necropsy findings. In some cases, confirmation was based on health treatment records or weighing records collected by the research team during routine farm visits. The farmers were asked to submit dead kids for post-mortem examination; when possible, the farmer also provided a clinical history and the suspected cause of death. If the necropsy could not be performed within 24 h of death, the carcass was refrigerated at 4 °C; otherwise, it was frozen at − 20 °C and thawed for 1 day before the examination. A systematic inspection of all thoracic and abdominal organs, tissues, joints, umbilicus, skull and brain was completed by a veterinarian. The cause of death was determined based on any gross pathological examinations or abnormal findings observed. Where necropsy did not reveal the cause of death, tissues from organs mentioned previously were fixed in 10% formalin, processed by a routine paraffin technique, sectioned at 5 µm. An analysis of the obtained specimens was processed by the research team at Grahamstown Veterinary laboratory. The farmers consistently cooperated with the research team throughout the research period. The sick and dead kids collected were grouped based on age (0–45 days, 46–90 days, 91–135 days and older than 136 days) to determine morbidity and mortality rates among various groups. Also, the time of exposure was grouped into seasons (post-rainy (March to May), cold-dry (June to August), hot-dry (September to November) and hot-wet (December to February). Reproductive losses such as abortion, stillbirths and dystocia were further grouped according to the parity of the doe (1st, 2nd, 3rd and 4th). Among various diseases, the proportional morbidity/mortality was calculated by the formula:

$$\begin{array}{c}\mathrm{Morbidity Rrate} (P)=\frac{\mathrm{Number of kids affected by a specific disease}}{\mathrm{Total number of kids affected by all the diseases}} \times X 100\\ \mathrm{Mortality rate }\left(P\right)=\frac{\mathrm{Number of kids died due to a specific disease}}{\mathrm{Total number of kids died due to all the diseases}}\times 100\end{array}$$

### Statistical analysis

Recorded data were entered in an excel spreadsheet as a database and used to analyse different attributable factors. The variables considered for the identified causes of morbidity and mortality were different climatic seasons of the year and mortality of kids to specific age group (birth to 45 days, 46 to 90 days, 91 to 135 days and older than 136 days). Parity of the doe (1st, 2nd, 3rd and 4th) was also used as a variable for reproductive losses. The data were analysed by Statistical Package for Social Science (SPSS) software 20.0 version, with goat kid as a unit of interest. Descriptive statistics were used to summarise the data. Chi-square (*χ*^2^) test was applied to test the existence of association and to see the level of significance between observed causes of mortality and associated risk factors, respectively, with significant difference being accepted at *P* < 0.05.

## Results

**Fig. 2:**
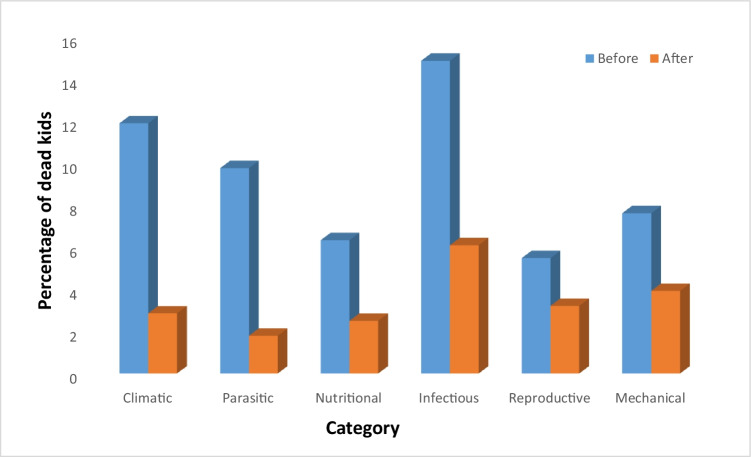
Overall mortality rate of kids according to various groups pre- and post-intervention

The current study recorded a total of 512 kids, with 235 kids being born prior introducing any intervention method (year-1), while the rest (277 kids) were enrolled during and post (year-2) introducing the intervention services to control disease outbreaks and mortality rate. Obtained results show that one hundred and thirty two kids died in year-1, accounting for 56.17%, while sixty two kids (22.38%) died in year-2. Several health problems were identified as potential causes of kid mortality requiring emergent attention. These health problems were categorized into several groups based on their causative agents (Fig. 2). Infectious diseases showed a notable decline in year-2 (6.38%) as opposed to year-1 (14.89%), while the climatic factors (11.92 vs 2.89%) and parasitic-related health problems showed a similar trend (9.79% vs 1.81%). Mechanical (7.66% vs 3.97%), reproductive (5.53% vs 3.25%) and nutritional (6.38% vs 2.53%)-related health problems also showed a slight decline.

Several diseases and parasitic infections have been presented in Table 1. A notable difference with regard to the prevalence of helminthosis was observed before and after introducing the intervention methods by the research team. Year-1 results show a consistent prevalence of helminthosis (*P* = 0.0058) in all the seasons, except during cold-dry season, with kids older than 90 days (*P* = 0.0019) as the most susceptible age group. On the other hand, helminthosis became less prevalent (*P* = 0.2588) in year-2, irrespective of the season while kids older than 90 days *P* = 0.0278) required further intervention. Ticks remained a problem throughout (*P* = 0.0069) the trial, except during cold-dry season, with kids older than 40 days as the most (*P* = 0.0004) affected age group. Diarrhoea was mostly prevalent (*P* = 0.0056) in kids younger than 40 days, while a decline (*P* = 0.0003) was observed after implementing the intervention methods by the research team. Kids belonging to age groups (0–40 days) and (41–90 days) were at higher risk (*P* = 0.006) of contracting contagious ecthyma, irrespective of the intervention status. On the other hand, less prevalent after the intervention of the research team. Kids younger than 90 days were at higher risk (*P* = 0.0044) of being injured by moving cars and predatory attacks as presented in Table 2. Gastro-intestinal-related deaths were recorded in year-1 but a significant decline (*P* = 0.0024) was noted after the intervention of the research team (Table 3).

**Table 1:** Prevalence of infections and parasites contributing to morbidity in goat kids according to climatic season and age group

	Type of ailment	Season				*χ*^2^ value	*P*-value	Age group (days)				*χ*^2^ value	*P*-value
		Post-rainy	Cold-dry	Hot-dry	Hot-wet			0–45	46–90	91–135	< 136		
Year-1	Helminthosis												
	Yes	26.67	0.00	15.15	16.67	12.54	0.0058	0.00	3.13	25.00	23.33	14.87	0.0019
	None	73.33	100.00	84.85	83.33			100.00	96.88	75.00	76.67		
	Ticks												
	Yes	9.52	22.22	33.33	25.00	6.49	0.09	3.33	18.75	23.08	40.00	12.16	0.0069
	None	90.48	77.78	66.67	75.00			96.67	81.25	76.92	60.00		
	Diarrhoea												
	Yes	50.00	22.22	18.18	20.83	12.61	0.0056	60.00	21.88	25.00	13.33	18.71	0.0003
	None	50.00	77.78	81.52	79.17			40.00	78.13	75.00	86.67		
	Contagious ecthyma												
	Yes	21.43	8.89	3.03	12.50	6.55	0.0875	13.33	31.25	5.77	0.00	17.52	0.006
	None	78.57	91.11	96.97	87.50			86.67	68.75	68.75	100.00		
	Footrot												
	Yes	0.00	8.89	6.06	0.00	5.68	0.1283	0.00	0.00	7.69	6.67	4.78	0.1883
	None	100.00	91.11	93.94	100.00			100.00	100.00	92.31	93.33		
Year-2	Helminthosis												
	Yes	0.00	15.79	10.53	18.18	4.03	0.2588	0.00	0.00	22.73	18.18	9.11	0.0278
	None	100.00	84.21	89.47	81.82			100.00	100.00	77.27	81.82		
	Ticks												
	Yes	9.09	26.32	36.84	45.45	6.45	0.0915	0.00	16.00	31.82	72.73	18.37	0.0004
	None	90.91	73.68	63.16	54.55			100.00	84.00	68.18	27.27		
	Diarrhoea												
	Yes	45.45	21.05	15.79	18.18	5.82	0.1208	53.85	32.00	18.18	0.00	10.06	0.0180
	None	54.55	78.95	84.21	81.82			46.15	68.00	81.82	100.00		
	Contagious ecthyma												
	Yes	0.00	0.00	5.26	0.00	2.78	0.4275	0.00	0.00	4.55	0.00	2.26	0.5204
	None	100.00	100.00	94.74	100.00			100.00	100.00	95.45	100.00		
	Footrot												
	Yes	0.00	10.53	0.00	0.00	5.63	0.1309	0.00	4.00	0.00	9.09	2.72	0.4362
	None	100.00	89.47	100.00	100.00			100.00	96.00	100.00	90.91		

Significant at *P* < 0.05.

*Year-1* before introducing the intervention effort, *Year-2* during and after introducing the intervention efforts.

**Table 2:** Prevalence of mechanical factors contributing to morbidity in goat kids according to climatic season and age group

	Type of ailment	Season				*χ*^2^ value	*P*-value	Age group (days)				*χ*^2^ value	*P*-value
		Post-rainy	Cold-dry	Hot-dry	Hot-wet			0–45	46–90	91–135	< 136		
Year-1	Predatory attacks												
	Yes	0.00	0.00	15.15	12.50	13.12	0.0044	3.33	3.13	5.77	10.00	1.78	0.6200
	None	100.00	100.00	84.85	87.50			96.67	96.88	94.23	90.00		
	Knocked by cars												
	Yes	21.43	11.11	9.09	8.33	3.64	0.3034	20.00	25.00	7.69	3.33	9.03	0.0289
	None	78.57	88.89	90.91	91.67			80.00	75.00	92.31	96.67		
Year-2	Predatory attacks												
	Yes	31.82	10.53	21.05	9.09	3.86	0.2776	30.77	28.00	13.64	0.00	5.30	0.1510
	None	68.18	89.47	78.95	90.91			69.23	72.00	86.36	100.00		
	Knocked by cars												
	Yes	13.64	15.79	10.53	9.09	0.39	0.9419	15.38	20.00	9.09	0.00	3.15	0.3691
	None	86.36	84.21	89.47	90.91			84.62	80.00	90.91	100.00		

Significant at *P* < 0.05.

*Year-1* before introducing the intervention effort, *Year-2* during and after introducing the intervention efforts.

**Table 3:** Prevalence of infectious diseases and management factors contributing to mortality in goat kids according to season and age group

	Cause of death	Season				*χ*^2^ value	*P*-value	Age group (days)				*χ*^2^ value	*P*-value
		post-rainy	cold-dry	hot-dry	hot-wet			0–45	46–90	91–135	< 136		
Year 1	GIT problems												
	Yes	0.00	25.00	16.67	30.00	14.41	0.0024	18.75	0.00	28.13	17.86	11.81	0.0080
	None	100.00	75.00	83.33	70.00			81.25	100.00	71.88)	82.14		
	Pneumonia												
	Yes	15.22	13.89	10.00	10.00	0.63	0.8904	34.38	7.50	9.38	0.00	18.70	0.0003
	None	84.78	86.11	90.00	90.00			65.63	92.50	90.63	100.00		
	External wound												
	Yes	4.35	13.89	10.00	15.00	2.83	0.4189	21.88	17.86	0.00	3.13	13.23	0.0042
	None	95.65	86.11	90.00	85.00			78.13	82.14	100.00	96.33		
	Heartwater												
	Yes	0.00	2.78	40.00	45.00	37.52	< 0.0001	0.00	0.00	31.25	42.86	33.13	< 0.0001
	No	100.00	97.22	60.00	55.00			100.00	100.00	68.75	68.75		
	Hypothermia												
	Yes	5.56	19.57	0.00	0.00	12.51	0.0058	15.63	7.50	3.13	7.14	3.45	0.3270
	None	94.44	80.43	100.00	100.00			84.38	92.50	96.88	92.86		
	Starvation												
	Yes	9.52	18.75	0.00	0.00	4.72	0.1937	15.38	7.69	10.00	0.00	2.45	0.4850
	None	90.48	81.25	100.00	100.00			84.62	92.31	90.00	100.00		
Year-2	GIT problems												
	Yes	0.00	14.29	15.38	16.67	2.79	0.4254	7.69	23.08	15.00	0.00	4.28	0.2325
	None	100.00	85.71	84.62	83.33			92.31	76.92	85.00	100.00		
	Pneumonia												
	Yes	0.00	9.52	0.00	0.00	4.03	0.2577	0.00	7.69	0.00	6.25	2.40	0.4937
	None	100.00	90.48	100.00	100.00			100.00	92.31	100.00	93.75		
	External wound												
	Yes	18.75	23.81	30.77	33.33	0.98	0.8056	26.92	22.73	3.77	6,67	15.59	0.0014
	None	81.25	76.19	69.23	66.67			73.08	17.53	96.33	93.33		
	Heartwater												
	Yes	28.57	0.00	53.85	50.00	12.52	0.0058	0.00	7.69	40.00	62.50	17.43	0.0006
	No	71.43	100.00	46.15	50.00			100.00	92.31	60.00	37.50		
	Hypothermia												
	Yes	0.00	31.25	100.00	0.00	15.64	0.0013	30.77	7.69	0.00	0.00	12.20	0.0067
	None	100.00	69.75	100.00	0.00			69.23	92.31	100.00	100.00		
	Starvation												
	Yes	9.52	18.75	0.00	0.00	4.72	0.1937	15.38	7.69	10.00	0.00	2.45	0.4850
	None	90.48	81.25	100.00	100.00			84.62	92.31	90.00	100.00		

Significant at *P* < 0.05.

*Year-1* before introducing the intervention effort, *Year-2* during and after introducing the intervention efforts.

Kids from all other age groups were at higher (*P* = 0.0080) risk of dying through gastro-intestinal problems than those in younger age group (0–45 days) in year-1. However, gastro-intestinal problems significantly declined in year-2, irrespective of the climatic season (*P* = 0.4254) and age group (*P* = 0.2325). Year-1 results show that high (*P* = 0.0003) proportion of kids belonging to the two age groups (0–45 days and 46–90 days) died due to pneumonia, irrespective of the season. Pneumonia became less prevalent in year-2 in both age groups (*P* = 0.2325) and season (*P* = 0.2577). Death due to external wounds remain a problem in both year-1 (*P* = 0.0042) and year-2 (*P* = 0.0014). Heartwater-related deaths remained a problem, irrespective of the intervention status (*P* < 0.0001), with high proportions recorded during hot-wet and hot-dry season. Kids older than 90 days were noted to be at higher risk (*P* < 0.0001) to heartwater-related deaths. Death due to hypothermia in year-1 was highly prevalent (*P* = 0.0058) during cold-dry season, with kids less than 40 days old as the sole victims. Mortality due to hypothermia became less prevalent (*P* = 0.0067) in year-2 after introducing the intervention efforts by the research team. Year-1 results show that starvation was only prevalent during cold-dry season than in other climatic seasons, with kids less than 40 days old as the most victimised age group compared to older age groups. However, year-2 showed a consistent decline on starvation-related mortalities. Dystocia, abortion and stillbirths were identified as the most dominant reproductive losses contributing to increased mortality rate (Table 4). Dystocia cases were insignificantly reported throughout the trial period, irrespective of the season and intervention status. Only does in their first parity (*P* = 0.0080) were at higher risk of experiencing dystocia compared to multiparous does. Stillbirths were another reproductive loss experienced by primiporous does before introducing the intervention efforts by the research team. Post-intervention results show a decline in stillborn cases, irrespective of the kidding season. Year-1 results show that abortion were mostly experienced by does in their first parity (*P* = 0.0101), while a significant decline (*P* = 0.0010) was noted after implementing the intervention efforts.

**Table 4:** Prevalence of reproductive fatalities contributing to mortality rate of goat kids according to climatic season and age group

	Cause of death	Season				*χ*^2^ value	*P*-value	Parity				*χ*^2^ value	*P*-value
		Post-rainy	Cold-dry	Hot-dry	Hot-wet			1st	2nd	3rd	4th		
Year-1	Dystocia												
	Yes	8.33	13.04	6.67	0.00	3.26	0.3528	27.50	0.00	0.00	0.00	27.60	< 0.0001
	None	91.67	86.96	93.33	100.00			72.50	100.00	100.00	100.00		
	Stillbirth												
	Yes	8.33	17.39	3.33	0.00	7.06	0.0699	30.00	0.00	0.00	0.00	30.36	< 0.0001
	None	91.67	82.61	96.67	100.00			70.00	100.00	100.00	100.00		
	Abortion												
	Yes	2.78	13.04	3.33	0.00	6.30	0.0977	20.00	0.00	0.00	0.00	19.59	0.0002
	None	97.22	86.96	96.67	100.00			80.00	100.00	100.00	100.00		
Year-2	Dystocia												
	Yes	4.76	12.50	0.00	0.000	3.31	0.3461	23.08	0.00	0.00	0.00	11.88	0.0078
	None	95.24	87.50	100.00	100.00			76.92	100.00	100.00	100.00		
	Stillbirth												
	Yes	4.76	0.00	0.00	0.00	1.98	0.5757	7.69	0.00	0.00	0.00	3.83	0.2803
	None	95.24	100.00	100.00	100.00			92.31	100.00	100.00	100.00		
	Abortion												
	Yes	3.51	14.52	2.33	0.00	11.33	0.0101	30.77	0.00	0.00	0.00	16.12	0.0011
	None	96.49	85.48	97.67	100.00			69.23	100.00	100.00	100.00		

Significant at *P* < 0.05.

*Year-1* before introducing the intervention effort, *Year-2* during and after introducing the intervention efforts.

## Discussion

A significant improvement in kids’ survival rate was recorded in the current study after introducing the intervention methods to control disease outbreaks and climate change-related deaths in communal goat flocks. In the last decade, community-based livestock improvement programs have gained attention as a promising approach to improve productivity of small ruminants in developing countries (Faruque et al., 2017). Such initiatives are seen as a potential solution to the disadvantaged communal goat production (Oyeyemi & Akusu, 2021). Notably, a decline in kid mortality in the current study implies that the project was a partial success and more community-based intervention projects should be encouraged. Nevertheless, practicality and implementation of such programs remain a challenge, mainly due to the issue of funding and participation of farmers (Alemnew et al., 2020). Various researchers claim that sustainability of livestock improvement programs in communal areas largely depends on farmers’ interest and participation. In most instances this is understood to be highly influenced by the socio-economic context of production (Sebei et al., 2004). These claims favourably agree with the observation made in the current study where it was suggested that any community-based intervention approach could play a vital role in improving the production status of communal goat flocks. Increased death rate of kids in year-1 in comparison to year-2 could be attributed to the negligence and reluctance to seek veterinary assistance as most farmers appeared to have accepted death of their goat kids as an unavoidable loss. Findings from this study agree with those reported by Sebei et al. (2004) who recorded a kid mortality rate of 46.51% due to poor animal health practices and lack of extension services.

Other health problems contributing to increased mortality rates in the current study include helminthosis, diarrhoea, tick loads, contagious ecthyma and Footrot. These disease conditions have also been reported in many countries worldwide (Arsoy, 2020; Talukder et al., 2020). Helminthosis was highly prevalent in all the climatic seasons, except during cold-dry seasons. An explanation could be that warmer temperatures and moist conditions favoured the growth of internal parasites (Slayi et al., 2014). Developing and implementing a specific deworming program for each kid per flock resulted to a decline in helminthosis prevalence in year-2. The programme targeted kids older than 90 days. The reason to deworm older kids is that the life cycle period for the internal parasite take 3 to 6 months to mature (Bélanger-Naud et al., 2021). It is alleged that deworming kids less than 3 months will be ineffective as the internal parasites will be undetected. Older kids got infected with helminthosis through grazing on infected pastures, while kids less than three months are still not allowed to join the flock (Fthenakis & Papadopoulos, 2018). Unfavourable conditions during cold-dry season resulted to less prevalence of helminth. Internal parasites species tend to delay their growth until favourable conditions arise later in the year (Faruque et al., 2017). This growth-delaying mechanism by various species of internal parasites is sometimes known as hypobiosis (Abraham et al., 2018; Adebambo et al., 2021).

Increased prevalence of diarrhoea in the current study may have been caused by bacteria, viruses, dietary factors and gastrointestinal parasites (Donkin & Boyazoglu, 2004; Yasmin et al., 2020). Death and sickness due to diarrhoea were reported in earlier works (Faruque et al., 2017; Oyeyemi & Akusu, 2021). An improvement in hygiene and careful husbandry of the kids in year-2 resulted to reduced or controlled diarrhoea even though it remained a matter of concern throughout the trial. Practicing consistent hygiene measures like provision of fresh water and cleaning the shelter and feeding materials should be encouraged. High tick loads recorded on kids during year-1 was due to browsing in heavily infested communal grazing areas and poor shelter hygiene. However, only older kids had higher tick counts compared to younger kids. The low-tick infestation in kids less than 45 days may be due to total confinement since they are not yet allowed to go together with their does, while older kids grazed on heavily infested pastures. A decline in tick load as recorded in year-2 could be attributed to various intervention strategies like a fortnight dipping program and collecting the manure every month. The practice of allowing manure to accumulate in animal houses result to increased tick loads, as it provides warmth and humidity that favour the proliferation of the ticks (Sebei et al., 2004). Predatory attacks remained a problem in year-1 as kids continuously graze without supervision of the herder. This has been a matter of concern even to previous studies conducted in the area. For instance, Slayi et al. (2014) also reported that kids were perceived to be at higher risk of getting attacked by jackals and hunting dogs. A decline in predatory attacks was noted after farmers were advised to hire some labour to collectively supervise the flocks while grazing in communal lands. Younger kids were at higher risk of getting knocked by cars, and the farmers were encouraged to house their kids or put them in a fenced garden to provide protection from stray dogs and cars. Similar works have been reported elsewhere in Africa (Bélanger-Naud et al., 2021; El-Abid & Abu Nikhaila, 2009).

Kids younger than 45 days were highly susceptible to pneumonia-related deaths in year-1. This finding is in agreement with those reported by Snyman (2010) who also noted high death rate of kids due to pneumonia in the first 35 days after birth. Pneumonia occurs when infectious and non-infectious agents cause the lungs of goats to become inflamed (Bhattarai, 2021; Vandana et al., 2015). The most frequent causes of respiratory infection and death are *Pasteurella multocida* or *Mannheimia haemolytica* (previously called *Pasteurella haemolytica*) (Alula et al., 2014; Diriba & Kebede, 2020). Heath complications from these pathogens are associated with poor management practices, such as overcrowded pens, poor housing conditions and other stressful conditions increasing susceptibility of the kids to *P. multocida* and *M. haemolytica pneumonias* (Arsoy, 2020; Yasmin et al., 2020). In most cases the pneumonia diagnosed after 2 months could be a complication arising from the weakening effects of earlier gastrointestinal problems. A timely treatment resulted to a decline in pneumonia death cases in year-2. Preventative measures included keeping kids in an open ventilated shelter as well as the addition of an ionophore to the feed, either monensin or lasalocid.

Season was defined to have a major influence on death rate due to heartwater in both year-1 and year-2. Heartwater-related deaths coincided with the increased tick loads as most fatalities occurred during hot-wet and hot-dry climatic seasons. Heartwater is transferred to goats through an infected tick bite (Mhlanga et al., 2018). An infected *Amblyomma hebraeum* is the tick species transmitting the heartwater in goats (Slayi et al., 2014). The seasonal occurrence of *A. hebraeum* has been studied before. It appears to be climate-dependent and varies throughout its distribution range (Vandana et al., 2015). The species has three-host life cycle, with larvae, nymphs and adults feeding on separate hosts (Debele et al., 2011). The adults are often visible in large numbers during warm-wet-summer months, larvae during colder, late autumn and winter months and nymphs during winter and spring months (Rashidi et al., 2011). Similar work has been documented by Sebei et al. (2004) who reported an increased mortality rate due to heartwater. Allowing animals to graze in communal lands infested with *Amblyomma* ticks species put the older kids at higher risk of contracting the disease. Implementing a holistic grazing management as well as burning the rangelands has been proven to be effective in controlling tick-on and off pastures. Death due to hypothermia remained a problem of interest in both year-1 than in year-2. Kids in their first 45 days of life were at noted to be at higher risk of being killed by extreme cold conditions in the area. Similar observation has been raised by Alula et al. (2014), who documented an increased death rate due to colds. An improved housing infrastructure resulted to better survival of young kids against climate extremes in year-2.

Starvation-related deaths to kids less than 45 days of age was a problem that farmers rarely considered in year-1. A high proportion of these deaths were experienced during cold-dry months of the year. Supplementing pregnant and lactating does in year-2 resulted to a decline in starvation-related deaths. This method became effective as most deaths were due to feed shortages which ultimately resulted poor mothering ability through the refusal of infant kids to suckle milk from their dams. Similar observations were made by Donkin and Boyazoglu (2004) in dairy goats. Dystocia remained a problem in both year-1 and 2, with does in their first parity as the only victim. Causes of dystocia can range from foetal factors such as large foetus or abnormal positioning (Adebambo et al., 2021). Other researchers highlighted maternal factors as the predisposing factor to dystocia (Hasan et al., 2015; Robertson et al., 2020). These factors include narrowed birth canal, lack of contractions or exhaustions for prolonged contractions (Moni & Samad, 2019). Consistent supervision to first-time does around parturition is highly advised to ease the problem. Stillborn kids were a problem to first-time does during year-1 and year-2, respectively. The actual cause for the increased prevalence of stillbirths remains unknown. However, it could be a genetic issue as most communal farmers tend to keep their bucks for longer periods thus opening chances of inbreeding (Chauhan et al., 2019). Stillbirths are usually born weak and die shortly after birth. Culling of bucks after every 3 years and outsourcing new sires from other regions are highly recommended to reduce the possibility of inbreeding depression in communal goat flocks. Developing some intervention methods targeting first-time mothers is a necessity. Abortion cases were mostly experienced by goats that are in their first parity during cold-dry season. The most important cause of abortion after day 90 of pregnancy is a nutritional energy deficiency caused by lack of good quality forage during dry months (Abraham et al., 2018). Lack of supplementary feeding to pregnant does could be blamed for this problem in year-1. Supplementation of pregnant goats and those in the first parity in year-2 could be commended for a slight decrease even though abortion remained a matter of concern.

## Conclusion

The current intervention project resulted to a decline in kid mortality rates due to various disease outbreaks and climate-related extremes. Obtained results imply that the approach was a moderate success. However, the affordability and ability to consistently maintain the program are a matter of concern as most farmers are underprivileged. This community engagement programme requires further investigation prior to implementing the intervention model at a broader level. So does the possibility that much could be gained by blending conventional drugs with traditional treatments to improve the survival rate of kids under village farming conditions. Formation of community-based animal health institutions to ensure consistent support to farmers is highly recommended.

## Data Availability

The data presented in this study is contained within the article.
